# What’s in a Name? How Useful is Current Rickettsial Taxonomy and Is Revision of Nomenclature Needed?

**DOI:** 10.4269/ajtmh.25-0459

**Published:** 2025-09-09

**Authors:** J. Stephen Dumler, David H. Walker

**Affiliations:** ^1^ Department of Pathology, School of Medicine, Uniformed Services University, Bethesda, Maryland, USA;; ^2^ Department of Pathology, University of Texas Medical Branch, Galveston, Texas, USA

Our understanding of rickettsial organisms, which is challenging due to their obligatory association with eukaryotic hosts, has benefited from advances with whole genomes.^1^^,^^2^ In this issue of the *American Journal of Tropical Medicine and Hygiene*, Paddock et al.^3^ extended genome studies of *Rickettsia* by modern analytical processes that show close taxonomic relationships between organisms that cause severe Rocky Mountain spotted fever and less virulent or nonpathogenic rickettsiae. The genetic relationships are undebated, but the taxonomic placement of *Rickettsia lanei sp. nov*. as a new species was not with scientific community consensus and was based on debatable principles.

## WHAT ARE THE MAJOR ISSUES IN *RICKETTSIA* TAXONOMY AND NOMENCLATURE?

Application of new names for disputable classifications led to a glut of *Rickettsia* species established using schemes that differ markedly from classification of their Alphaproteobacteria relatives and other prokaryotes. This led to significant confusion among physicians and clinical microbiologists who have difficulty discerning the value of nomenclature utilizing criteria unlinked to rickettsial virulence mechanisms. The use of this systematics approach has been long justified by many who hold the certifying belief of “valid publication”, a term for registering names regulated by the International Union of Microbiological Societies and the International Code of Nomenclature of Bacteria. While valid publication has an important role, neither the naming process nor “valid publication” sanction phylogenetics/genomics or taxonomy, but simply assure that names are properly documented, clearly defined, and conform to standardized rules. Valid publication does not assure scientific accuracy or consensus regarding the taxa designations – this is the purview of scientific debate. As stated in the List of Prokaryotic Names with Standing in Nomenclature, “There is no official classification of prokaryotes, but the *names* given to prokaryotes are regulated.”^4^

## HOW DID CURRENT RICKETTSIAL NOMENCLATURE DEVELOP?

Because rickettsiae are obligately intracellular bacteria, the determination of phenotypic and chemotaxonomic characteristics is challenging; thus, the focus on phylogenetics/phylogenomics. Widely accepted prokaryote species criteria include digital DNA-DNA hybridization (dDDH) thresholds of 70%, and 79% for subspecies.^5^^,^^6^ However, the criteria for *Rickettsia* species are distinct and arguable, leading to confusion and ineffective communication regarding disease, treatments, virulence and pathogenicity, and for reporting laboratory results, interpreting data, and informing appropriate public health measures.^7^ Imagine the confusion that the currently recommended criterion for *Rickettsia* species (dDDH <92.30%)^8^ would wreak on *Escherichia*. Unlike *Escherichia*, where virulence is often tied to established genetic components, independent of our understanding of evolution, this approach leads to confusing subdivisions for *Rickettsia,* undermining the purposes of nomenclature, which is to provide an unambiguous system for naming organisms that reduces confusion and provides a common language for clear and precise communication. That this is true can be gleaned from the expanse of newly named species in the spotted fever group of *Rickettsia*, and the extraordinarily large number of *Candidatus* species lacking a bacterial isolate.

Taxonomy origins for *Rickettsia* can be traced to studies using mouse immune sera that in 1969 Ormsbee described to include four distinct groups of spotted fever rickettsiae: “*R. rickettsii*/*R. sibirica”* group; “*R. conori*/*R. parkeri”* group; “*R. australis*/*R. akari”* group, and “*R. montana* and Western Montana U” group.^9^ By 1978, mouse serotyping of heterotypic strains showed similarities and differences that suggested 12 distinct groups.^10^ It was this test that formed the basis of modern spotted fever group *Rickettsia* taxonomy and nomenclature. However, this serotyping method was subsequently determined to be based primarily on rickettsial outer membrane protein A (OmpA) and OmpB.^11^

While advances in prokaryotic, archaeal, and eukaryotic phylogeny and taxonomy were rapid, defining taxonomic boundaries for the genus *Rickettsia* was a major challenge. This included difficulties of cultivating rickettsiae; biosafety concerns; slow growth; lack of robust genetic tools; a reduced genome; significant antigenic and genetic overlap; highly similar phenotypic attributes; and unclear markers of pathogenicity, virulence, and disease causation. The use of PCR was helpful in identifying infection absent cultivation but also led to taxonomic and nomenclature controversies given the reliance on serotyping methods for speciation.

The use of multilocus sequence typing (MLST) allowed taxa to be established by gene amplification and Sanger sequencing as estimates onto which nomenclature could be assigned. Thus, Fournier et al. published a taxonomic method that used MLST calibrated against *Rickettsia* mouse serotyping.^12^ In this work, very close relationships between named species (e.g. *R. rickettsii*, *R. sibirica*, *R. conorii*, and *R. parker*i) were accepted as defining species differences, creating a nomenclature system now incongruent with other Alphaproteobacteria. Lacking cultivated isolates but with readily available tools, this started the “discovery” of new *Rickettsia* species. At a time when few *Rickettsia* genomes were available, interrogating 1.5% of the genome was considered valuable. However, as of July 2025, more than 165 publications in PubMed and over 529 in Google Scholar cited this work. In those in Google Scholar, 21 new *Candidatus* species were proposed in part justified by the MLST approach. Similarly, current NCBI Taxonomy lists 39 new *Candidatus* species, 38 “established” species, as well as 53 subspecies, and 255 unclassified spotted fever group names.^13^

## WHOLE GENOME SEQUENCING FOR *RICKETTSIA* SYSTEMATICS?

With whole genome sequencing, taxonomy and nomenclature for the families Rickettsiaceae and Anaplasmataceae were revisited. Chung et al.,^14^ examined core genome sequence alignment identities and revealed that among 782 pairwise comparisons of bacterial genomes classically defined as different species, the majority would be classified as the same species, with all but 10 “re-classifications” in the genus *Rickettsia.* Additionally, 27 species of *Rickettsia* could be consolidated into 9 species, and of 46 spotted fever *Rickettsia* genomes, 44 could be within a single species.^14^ A subsequent method was proposed by authors of the Rickettsiales MLST schema, using an approach similar to Chung et al., that demonstrated nearly identical phylogenomic trees.^8^^,^^12^^,^^14^ However, the authors argued that “The strictly intracellular lifestyle, few measurable phenotypic properties and low genetic heterogeneity make current standard genomic criteria for bacterial species definition inapplicable to *Rickettsia* species”. Thus, calibrated by establishing dDDH and ANI cutoffs to replicate the divisions of the MLST approach, the result predictably confirmed the MLST work.^8^

Another proposed approach examined 1,104 “trusted” type strain whole genomes in Alphaproteobacteria.^15^ Here, the authors investigated species and subspecies boundaries by dDDH and gene content, applying accepted cutoffs of 70% for species and 79% for subspecies.^5^^,^^6^ This revealed that several orders, families, genera, and species needed revision, significantly including the Rickettsiales. Emendations were offered that would collapse the number of spotted fever group *Rickettsia* species to create five new subspecies of *R. conorii* that were previously validly published species.^15^ A separate reanalysis of all spotted fever *Rickettsia* genomes using this approach reduced the group from 30 to as few as four species, and as many as 18 new subspecies that could include the majority of human pathogens within the single species *Rickettsia rickettsii*. This is reflective of more recent genome classifications such as *R. rickettsii* subspecies *californica* (modest to moderate severity),^16^ and likely *R. lanei sp. nov*. (severe disease). Remarkably, the phylogenetic tree structures created in all three of these publications are nearly identical. The key difference is how the classifications of species and subspecies were decided.

## WHAT IS THE PROBLEM AND THE POTENTIAL SOLUTION?

Accordingly, the problem with spotted fever group *Rickettsia* classification is related directly to nomenclature assignments. The science is the same, but the names are different. Phylogeny explains how bacteria are related, and taxonomy is their systematic arrangement into related groups. In contrast, nomenclature involves the assignment of names to the taxa in accordance with international rules. Valid publication establishes precedents to avoid arbitrary name changes and to provide consistency that promotes clear communication among users. How a genus, species, or subspecies is established is not decided by nomenclature; that only communicates what has been presented and hopefully accepted by consensus. Unfortunately, that consensus has not been achieved among rickettsiologists. This has led to extensive confusion among key information users, largely health care practitioners and the public.

The outstanding work of Paddock et al.^3^ furthers understanding of the phylogenomic position of an important highly pathogenic bacterium, but the classification schema applied promotes rather than lessens confusion. Fortunately, there is a mechanism to resolve these differences and to achieve consensus such that nomenclature can work for all interested parties. This involves cooperative discussion about the strengths and weaknesses with all approaches, establishing clear goals and timelines for achievement, and dissemination of the opinions of all parties involved in resolving the issues to mutual satisfaction. The way forward is to establish, with international participation and broad representation of disciplines and interests, a subcommittee on Rickettsiales taxonomy and nomenclature, guided by policies and bylaws of the International Committee on Systematics of Prokaryotes. This approach could address many concerns to provide a unified nomenclature that addresses the needs of all interested parties.
